# Analysis of individual patient data to describe the incubation period distribution of Shiga-toxin producing *Escherichia coli*

**DOI:** 10.1017/S0950268819000451

**Published:** 2019-03-27

**Authors:** A. Awofisayo-Okuyelu, I. Hall, E. Arnold, L. Byrne, N. McCarthy

**Affiliations:** 1 Department of Zoology, University of Oxford, Oxford, United Kingdom of Great Britain and Northern Ireland; 2 National Institute of Health Research, Health Protection Research Unit (NIHR HPRU) in Gastrointestinal Infections, University of Liverpool, Liverpool, United Kingdom of Great Britain and Northern Ireland; 3 School of Mathematics, University of Manchester, Manchester, United Kingdom of Great Britain and Northern Ireland; 4 National Infection Service, Centre for Infectious Disease Surveillance and Control, Public Health England, London, United Kingdom of Great Britain and Northern Ireland; 5 Department of Medicine, University of Warwick, Coventry, United Kingdom of Great Britain and Northern Ireland

**Keywords:** Analysis of data, incubation period, individual patient data analysis, Shiga-like toxin-producing *E. coli*

## Abstract

Shiga-toxin producing *Escherichia coli* (STEC) is a pathogen that can cause bloody diarrhoea and severe complications. Cases occur sporadically but outbreaks are also common. Understanding the incubation period distribution and factors influencing it will help in the investigation of exposures and consequent disease control. We extracted individual patient data for STEC cases associated with outbreaks with a known source of exposure in England and Wales. The incubation period was derived and cases were described according to patient and outbreak characteristics. We tested for heterogeneity in reported incubation period between outbreaks and described the pattern of heterogeneity. We employed a multi-level regression model to examine the relationship between patient characteristics such as age, gender and reported symptoms; and outbreak characteristics such as mode of transmission with the incubation period. A total of 205 cases from 41 outbreaks were included in the study, of which 64 cases (31%) were from a single outbreak. The median incubation period was 4 days. Cases reporting bloody diarrhoea reported shorter incubation periods compared with cases without bloody diarrhoea, and likewise, cases aged between 40 and 59 years reported shorter incubation period compared with other age groups. It is recommended that public health officials consider the characteristics of cases involved in an outbreak in order to inform the outbreak investigation and the period of exposure to be investigated.

## Introduction

Shiga-toxin producing *Escherichia coli* (STEC) infection is a zoonotic pathogen that causes gastrointestinal illness. The main routes of transmission are foodborne, direct or indirect animal contact, and person to person spread [1]. Common symptoms include diarrhoea, nausea and vomiting [2] and some cases report symptoms of haemorrhagic colitis (HC), including bloody diarrhoea and abdominal pain [3]. Between 6% and 14% of cases develop haemolytic uraemic syndrome (HUS), a serious complication of STEC infection [4].

Outbreaks of STEC are frequently reported in England and Wales, with an average of 13 outbreaks reported annually between 2009 and 2016 [5]. Investigating outbreaks, which contributes to reducing the burden of STEC infections, usually involves collecting information on risk factors through patient interviews and questionnaires, either as part of routine surveillance or through epidemiological studies and/or environmental investigations. The information is collected for a set time period prior to clinical illness, aiming to cover the period a case may have been exposed to the pathogen. Conducting interviews during an outbreak investigation should be informed by a good understanding of when exposures leading to disease are likely to have occurred in order to support effective and efficient investigation.

Incubation period is the time between exposure to the infecting pathogen and the onset of clinical illness. Accurate knowledge of this parameter is useful in narrowing down the possible time of exposure, excluding secondary cases and also declaring the end of an outbreak [6]. It is also useful in understanding the pathogenesis of STEC and estimating the possible extent of the spread of infections. In the large notable STEC O104 outbreak in Germany [7], the incubation period was longer than expected resulting in challenging epidemiological investigations particularly when collating relevant exposure details.

Despite its importance, available reports on the incubation period of STEC are conflicting, such as the differing expected distributions proposed by the World Health Organization and the Centre for Disease Control and Prevention of 3–8 days and 3–4 days, respectively [8, 9]. Additionally some outbreaks have reported incubation periods of a median of 9 days [10]. Incorrect estimations of the incubation period may result in formulating inaccurate case definitions and wrongly defining exposure times.

Systematic reviews of outbreak reports and experimental studies have proved invaluable in describing the incubation period distribution of gastrointestinal pathogens, and identifying possible influencing factors [11, 12]. However, some patient factors, such as patient demographics and reported symptoms, could not be examined as they were not available in published outbreak reports at the individual level. Analysing individual patient data (IPD) provides an avenue to study these additional factors. IPD can be extracted from both sporadic cases and cases associated with outbreaks. The source of infection is more likely to be reliably identifiable in outbreaks than for sporadic cases, making these data a useful resource to study incubation period. Unusually, England has a national system to collate individual case data for STEC cases and outbreaks allowing analyses of these data.

We have extracted IPD from outbreaks with a reported source of infection reported in England and Wales between 2009 and 2016. We calculated the incubation period for each case and analysed both patient and outbreak data to identify the distribution of incubation periods and factors that may be associated with the incubation period of STEC.

## Methods

### Case ascertainment

Routine enhanced surveillance of STEC has been ongoing in England and Wales since 1 January 2009 [13]. The local laboratories are expected to report presumptive isolates to Public Health England Centres (PHEC). As part of a routine follow-up, the PHEC administers a standardised enhanced surveillance questionnaire (ESQ) [14] to each patient. Clinical and epidemiological data collected by the ESQ include demographic information; relevant occupation including food handlers, health care workers and child carers; date of illness onset; clinical condition including HUS progression; details of potential exposures including travel, food and water consumption, contact with animals and environmental factors; and outbreak status. In addition, cases are required to indicate the symptoms they experienced from a standard set of symptoms. Completed questionnaires are then forwarded to the gastrointestinal department of Public Health England (PHE) and entered into the National Enhanced STEC Surveillance System.

Cases associated with outbreaks where the source was identified were included in the study. Additional outbreak information such as outbreak reports and outputs of investigations were also sought and cases were included where there was information at the outbreak or individual level allowing identification of their exposure date.

### Comparing characteristics of excluded and included cases

The cases excluded from the study were described according to available characteristics including age, gender, ethnicity, geographical region of outbreak, reported symptoms and mode of transmission. A *χ*^2^ test for equality of proportions was calculated to compare the distribution of a given variable amongst cases in the excluded group and cases in the included group. The significance level was set at 0.05.

### Microbiological methods

Confirmation and serotyping of STEC was done at the PHE Gastrointestinal Bacteria Reference Unit. Strains of serogroup O157 were further differentiated by phage type (PT) [13].

### Calculation of incubation period

Date of illness onset, for the purpose of this study, was defined as the first day of symptoms as reported by the patient, which is routinely collected in the ESQs.

The date of exposure was defined as the day the patient reported to have come into contact with the identified source of infection, such as a contaminated food item or infected animal. The date of exposure is not asked as a routine question on the ESQ, but rather, the question is asked as to whether exposure occurred during the 7 days before illness. Where the exposure date was unavailable on the ESQ, additional data sources were sought, such as reports of outbreak investigations and details of epidemiological studies conducted, in order to deduce the reported date of exposure.

The incubation period was calculated as the difference in days between the reported date of symptom onset and the date of exposure. Where either or both dates were unknown, the case was excluded from the study.

### Descriptive analysis

Frequencies and percentages were calculated to describe the cases and outbreaks. Cases were described according to the reported characteristics including age, gender, ethnicity, geographical region of residence, infecting PT and reported symptoms. Outbreaks were described according to mode of transmission, PT and geographical region of outbreak.

### Regression analyses

In order to identify factors that may predict the distribution of the incubation period, we examined the relationship between the patient and outbreak characteristics and incubation period using a multi-level regression (MLR) model. The regression model included the patients’ characteristics, the mode of transmission and region of outbreak as explanatory variables.

We first examined the association between incubation period and each variable in a univariate analysis. Where a significant association was observed (*P* = 0.10), we included these variables in a multivariable model. A final model was developed using a backward step-wise procedure and the significance level was set at 0.10.

The model was fitted to log-transformed data to account for skewness, however, in order to check for model sensitivity, we investigated alternative transforms on the data. The additional models we evaluated were a linear MLR model using untransformed data and a generalised MLR model using the *γ* family.

All analyses were conducted using the statistical software R version 3.2.3 (2015-12-10).

## Results

A total of 106 STEC outbreaks were reported in England and Wales during the study period of 2009–2016 affecting 1459 cases. The source of infection was unknown for 42 of the 106 outbreaks and these were excluded.

**Fig. 1.**
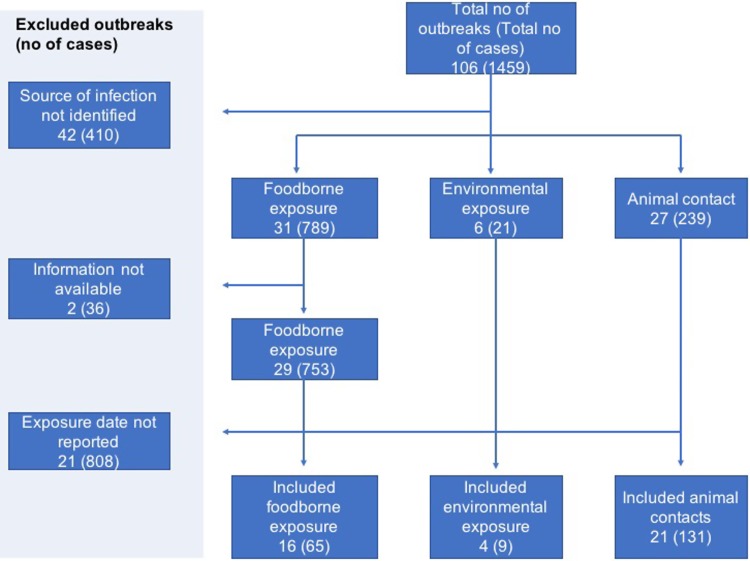
Flowchart showing selection of outbreaks and cases with available individual data.

In two foodborne outbreaks, details of the investigation which included patient information and dates of exposure could not be retrieved, hence these outbreaks were excluded. Reviewing the outbreak information available on the remaining 62 outbreaks and searching through the data reported by the affected 1013 cases, dates of exposure were identified for 205 cases in 41 outbreaks (Fig. 1).

### Comparing characteristics of excluded and included cases

The characteristics of cases excluded from the study were significantly different from those included in the study (Supplementary Table S1). Distribution of cases according to age group (*P*-value < 0.0001), ethnicity (*P*-value < 0.0001), reported symptoms (*P*-value < 0.05) and mode of transmission (*P*-value < 0.0001) were significantly different in both groups of cases. All included cases were of serotype O157.

### Description of outbreaks

Of the 41 outbreaks included in the study, 51.2% (21/41) were associated with animal contact through contact with livestock during farm visits, 39% (16/41) were foodborne and 9.8% (4/41) were associated with environmental exposures, some of which included an outdoor sporting activity and animal fair. Of the 16 foodborne outbreaks, red meat or salad were the implicated food vehicles in 50% and 18.8% of outbreaks, respectively (Table 1).

**Table 1.** Characteristics of outbreaks included in the analysis

Variable	Number of outbreaks (*N* = 41); (%)
Mode of transmission	
Animal contact	21 (51.2)
Environmental exposure	4 (9.8)
Foodborne exposure	16 (39.0)
Geographical region of outbreak	
East Midlands	3 (7.3)
East of England	3 (7.3)
National	4 (9.8)
North East	2 (4.9)
North West	7 (17.1)
South East	5 (12.2)
South West	7 (17.1)
Wales	1 (2.4)
West Midlands	1 (2.4)
Yorkshire and Humber	8 (19.5)
Phage type	
PT 21/28	15 (36.6)
PT 8	7 (17.1)
PT 2	5 (12.2)
PT 1	2 (4.9)
PT 32	2 (4.9)
PT 4	2 (4.9)
PT 54	1 (2.4)
PT 34	1 (2.4)
RDNC	1 (2.4)
Mixed PT outbreaks	4 (9.8)
Unknown	1 (2.4)

Most of the outbreaks were localised, occurring in the Yorkshire and Humber (19.5%; 8/41), South West (17.1%; 7/41) and North West regions (17.1%; 7/41) of England. No outbreaks occurred in London only, however 84.3% (27/32) of the outbreak cases reported in London were associated with a particular outbreak in the South East. Four national outbreaks occurred, associated with nationally distributed products.

The most common PT causing outbreak was PT 21/28 accounting for 36.6% (15/41) of the outbreaks. PT 8 accounted for 17.1% (7/41) and there were four outbreaks with mixed PTs.

**Fig. 2.**
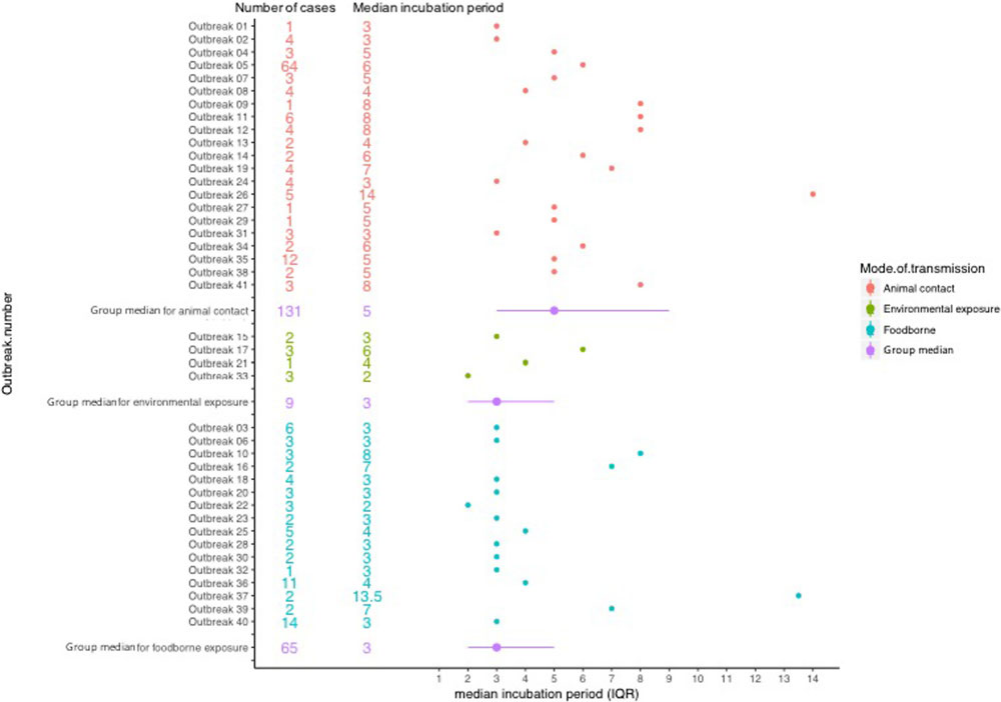
Forest plot showing median incubation period and IQR of outbreaks included in the study.

The median incubation period of outbreaks ranged from 2 days, in an outbreak associated with environmental exposure, to 13.5 days in a foodborne outbreak (Fig. 2).

### Description of cases

A total of 205 cases were included in the study, of which 57.1% (117/205) were children under 10 years of age. Females accounted for 59% (121/205) of cases (Table 2). Serotyping results were available for all cases, all of which were serotype O157. Phage typing results were available for 204 cases and PT 21/28 was most commonly reported (65.4%; 134/205) (Table 2).

**Table 2.** Characteristics of study population in the analysis of individual patient data of STEC

Variable	Number of cases (*N* = 205)	Percentage (%)	Mean incubation period (days)	Median incubation period (days)
Age group				
0–4 years	69	33.7	6.0	6.0
5–9 years	48	23.4	6.9	5.0
10–19 years	23	11.2	5.1	4.0
20–29 years	26	12.7	5.2	3.5
30–39 years	16	7.8	5.9	4.0
40–59 years	10	4.9	3.2	2.0
60 and above	13	6.3	4.3	4.0
Gender				
Females	121	59.0	6.2	4.0
Males	84	41.0	5.5	4.0
Ethnicity				
White	166	81.0	5.9	4.0
Mixed ethnicity	3	1.4	6.0	5.0
Black/Black British	1	0.5	2.0	2.0
Chinese	1	0.5	7.0	7.0
Unknown	34	16.6		
Relevant occupation				
Food handler	8	3.9	5.2	4.5
Healthcare worker	4	2.0	5.5	4.5
Childcare workers	40	19.5	7.3	6.0
Non-relevant occupation or children	153	74.6		
Geographical region				
East Midlands	4	1.9	3.7	3.0
East of England	17	8.3	8.7	5.0
London	32	15.6	7.5	6.5
North East	27	13.2	3.7	3.0
North West	27	13.2	4.6	4.0
South East	45	21.9	5.0	4.0
South West	16	7.8	5.1	4.0
Wales	1	0.5	6.0	6.0
West Midlands	9	4.4	9.3	8.0
Yorkshire and Humber	27	13.2	6.7	4.0
Phage type				
PT 21/28	134	65.4	6.2	5.0
PT 8	18	8.8	4.4	3.0
PT 1	17	8.3	5.2	4.0
PT 2	14	6.8	3.7	3.0
PT 54	6	2.9	9.5	11.0
PT 32	5	2.4	4.6	4.0
PT 34	3	1.5	6.0	6.0
PT 4	3	1.5	3.7	5.0
PT 31	2	1.0	13.5	13.5
RDNC	2	1.0	5.5	5.5
Unknown	1	0.5		
Reported symptoms				
HUS^a^	17	8.3	5.2	5.0
Diarrhoea	191	93.2	5.8	4.0
Bloody diarrhoea	135	65.9	4.8	4.0
Nausea	102	49.8	5.6	4.0
Vomiting	87	42.4	5.2	4.0
Abdominal pain	164	80.0	5.6	4.0
Fever	57	27.8	5.3	4.0
Mode of transmission				
Direct animal contact	131	63.9	6.9	5.0
Environmental exposure	9	4.4	3.6	3.0
Foodborne	65	31.7	4.1	3.0

a Haemolytic uraemic syndrome.

Foodborne transmission accounted for 31.7% (65/205) of cases while 63.9% (131/205) of cases were attributed to direct animal contact. There was a median of three cases per outbreak for each group of transmission; however, the maximum number of cases reported varied by mode of transmission. Transmission through animal contact included one large outbreak with 64 cases [15], which was the largest number reported, and the largest outbreak of foodborne transmission reported 14 cases with identifiable incubation times.

Twenty-one per cent (45/205) of cases lived in the South East region of England, and of these, 80% (36/45) were cases associated with animal contact exposures. All but one of the cases living in London were associated with animal contact (96.9%; 31/32).

### Reported symptoms

**Fig. 3.**
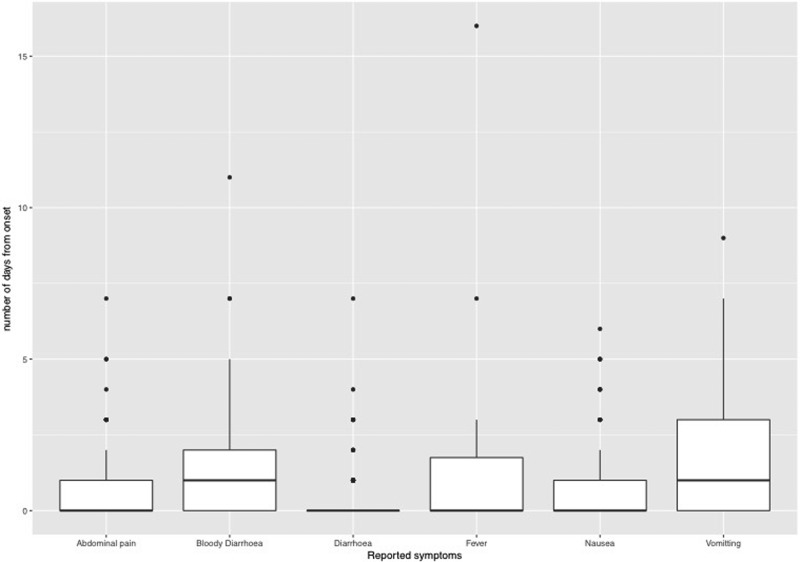
Boxplot showing median days and interquartile range between onset of first symptom and reported symptoms.

Cases reported a combination of symptoms and the most frequently reported symptoms were diarrhoea (93.2%), abdominal pain (80%), bloody diarrhoea (65.9%) and nausea (49.8%). The median time interval between the onset of illness and specific symptoms was 0 days for diarrhoea, nausea, fever and abdominal pain and 1 day each for bloody diarrhoea and vomiting (Fig. 3).

### Incubation period and associated factors

**Fig. 4.**
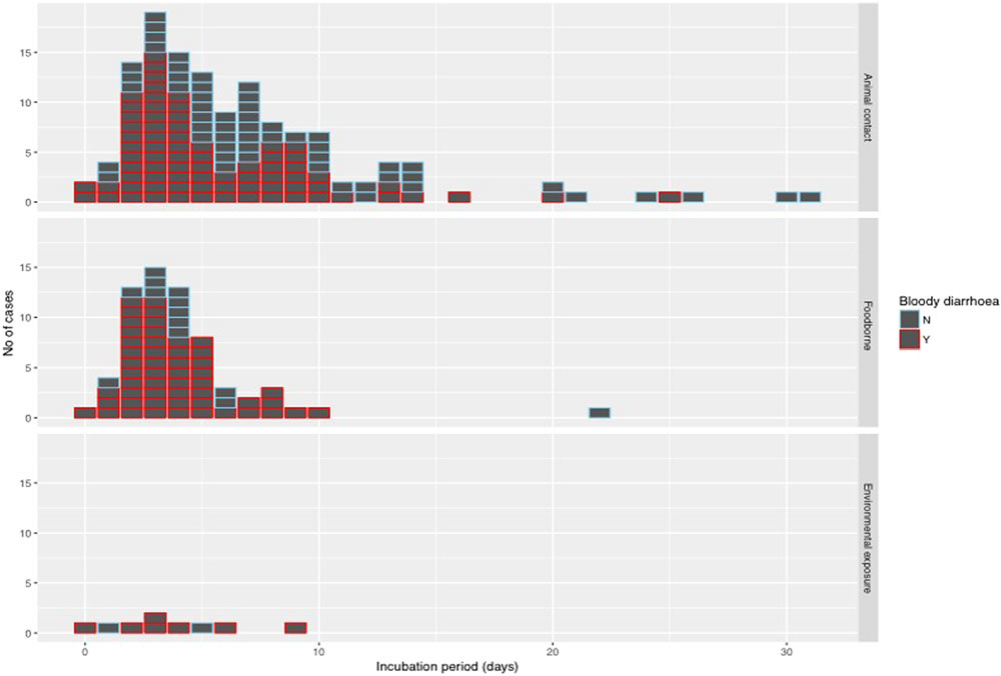
Histogram of reported incubation period by mode of transmission and indicating cases with bloody diarrhoea.

The median incubation period was 4 days with an interquartile range of 3–7 days. Just over 10% (22/205) of cases reported an incubation period longer than 10 days (Fig. 4). With the exception of one case of foodborne infection with an incubation period of 22 days, long incubation periods of more than 10 days were observed in 21 cases acquiring their infection from animal contact.

Using the log transformed data, the univariate analysis identified reporting bloody diarrhoea, cases aged 40–59 years and mode of transmission as variables that were significantly associated with the duration of incubation period, while reporting vomiting showed a weak association (*P*-value of 0.1) but met the inclusion criteria to be included in the multivariable model (Table 3). In the final multivariate analysis, reporting bloody diarrhoea and cases aged 40–59 years old remained significantly associated with incubation period (*P*-values of <0.01 and 0.01, respectively). The geometric mean ratio of cases with bloody diarrhoea *vs.* cases without bloody diarrhoea was 0.76 (95% CI 0.63–0.91) indicating that cases reporting bloody diarrhoea had an incubation period 24% shorter on average (Table 3). This finding was dependent on inclusion of the data from the 64 people in the largest animal contact outbreak (outbreak 05 in Fig. 2), in which 46% of cases reported bloody diarrhoea compared with 74% from the other outbreaks in the study. This significant association was not lost when analysing data excluding outbreak 05 (*P*-value = 0.01) and analysing only data from outbreak 05 (*P*-value = 0.05).

**Table 3.** Multi-level regression model showing factors associated with incubation period

	Model 1 (univariate model)		Model 2 (full multivariate model)		Final model	
Variable	Geometric mean ratio (95% confidence interval)	*P*-value	Geometric mean ratio (95% confidence interval)	*P*-value	Geometric mean ratio (95% confidence interval)	*P*-value
Age group						
0–4 years	1.00 (Reference)		1.00 (Reference)		1.00 (Reference)	
5–9 years	1.06 (0.85–1.32)	0.60	1.09 (0.90–1.38)	0.43	1.09 (0.88–1.34)	0.45
10–19 years	0.85 (0.63–1.13)	0.27	0.91 (0.70–1.26)	0.54	0.89 (0.66–1.17)	0.40
20–29 years	0.89 (0.66–1.19)	0.41	0.97 (0.75–1.36)	0.86	0.94 (0.70–1.06)	0.67
30–39 years	0.89 (0.66–1.24)	0.48	0.95 (0.70–1.39)	0.78	0.91 (0.65–1.25)	0.55
40–59 years	0.57 (0.37–0.85)	<0.01	0.64 (0.45–1.06)	0.04	0.61 (0.41–0.91)	0.01
60 and above	0.83 (0.57–1.19)	0.31	0.90 (0.64–1.37)	0.58	0.87 (0.60–1.25)	0.46
Gender						
Females	1.00 (Reference)					
Males	0.96 (0.81–1.13)	0.60				
Geographical region of outbreak						
East Midlands	1.00 (Reference)					
East of England	0.67 (0.33–1.27)	0.27				
National	0.85 (0.44–1.52)	0.63				
North East	0.68 (0.34–1.26)	0.30				
North West	0.62 (0.35–1.06)	0.12				
South East	0.70 (0.40–1.23)	0.25				
South West	0.77 (0.42–1.35)	0.41				
Wales	0.84 (0.23–2.99)	0.80				
West Midlands	0.56 (0.20–1.51)	0.30				
Yorkshire and Humber	0.82 (0.47–1.39)	0.52				
Phage type						
PT 1	1.00 (Reference)					
PT 2	0.78 (0.48–1.29)	0.36				
PT 21/28	0.96 (0.65–1.44)	0.83				
PT 31	2.33 (0.94–6.03)	0.09				
PT 32	1.00 (0.52–1.97)	0.99				
PT 34	1.21 (0.54–2.97)	0.66				
PT 4	0.78 (0.36–1.73)	0.55				
PT 54	1.60 (0.84–3.14)	0.19				
PT 8	0.93 (0.59–1.50)	0.77				
RDNC	1.26 (0.51–3.09)	0.63				
Reported symptoms						
HUS						
No	1.00 (Reference)					
Yes	0.87 (0.64–1.17)	0.34				
Diarrhoea						
No	1.00 (Reference)					
Yes	0.97 (0.70–1.35)	0.87				
Bloody diarrhoea						
No	1.00 (Reference)		1.00 (Reference)		1.00 (Reference)	
Yes	0.75 (0.62–0.89)	<0.01	0.78 (0.64–0.93)	0.01	0.76 (0.63–0.91)	<0.01
Nausea						
No	1.00 (Reference)					
Yes	0.91 (0.77–1.08)	0.29				
Vomiting						
No	1.00 (Reference)		1.00 (Reference)			
Yes	0.89 (0.75–1.05)	0.17	0.93 (0.79–1.11)	0.45		
Abdominal pain						
No	1.00 (Reference)					
Yes	0.90 (0.73–1.11)	0.34				
Fever (0.6)						
No	1.00 (Reference)					
Yes	0.96 (0.79–1.16)	0.66				
Mode of transmission						
Direct animal contact	1.00 (Reference)		1.00 (Reference)			
Foodborne	0.80 (0.63–1.01)	0.05	0.89 (0.68–1.05)	0.37		

From the sensitivity analysis, testing alternative data transformations had no effect on the results.

## Discussion

The mean and median incubation period observed in our study were 6 and 4 days, respectively, with an interquartile range of 3–7 days, within the range reported in a systematic review of the incubation period of STEC where the means of subgroups with limited evidence of heterogeneity ranged between 3.5 and 8.1 days (Awofisayo-Okuyelu *et al.* Accepted – Epidemiological Reviews).

Our regression analysis showed that cases reporting bloody diarrhoea have on average shorter incubation period than cases not reporting this symptom. This association was not lost when data excluding outbreak 5 was analysed and also when data from only outbreak 05 was analysed.

HC and the associated symptoms of bloody diarrhoea and abdominal pain is an afebrile syndrome of acute STEC infection [16]. The ability of the bacteria to adhere to the intestinal mucosa and produce cytotoxins contributes to its virulence factors [17]. Although the bloody diarrhoea is itself among the later symptoms, it can be suggested that the virulence factors of the bacteria may be associated with both bloody diarrhoea and the early pathogenesis of the disease leading to earlier onset of other symptoms.

According to our study, cases belonging to the age group 40–59 years were significantly associated with shorter incubation period, unlike other studies where children have reported shorter incubation periods [18–20]. Likewise, results from a systematic review on the incubation period of STEC identified outbreaks involving mostly adults as having longer incubation periods, although, this was particular to cases of one non-O157 serotype (O104) (Awofisayo-Okuyelu *et al.* Accepted – Epidemiological Reviews).

We found no association of incubation time with pathogen characteristics (PT) or development of HUS. In England and Wales, serotype O157 is most commonly associated with human disease [13], and also commonly implicated in outbreaks [5]. However, there is a diagnostic bias for detection of O157 making it more likely to be investigated than other STEC serotypes. This may account for the difference between excluded and included cases where all non-O157 cases were excluded from the study as information on time of exposure was unavailable. We cannot therefore comment on differing incubation periods across serotypes, with some published evidence that, e.g. serotype O104 infections may have a particularly long incubation period [7]. According to Byrne *et al.* [13], the three most commonly reported STEC O157 PTs in England as at 2012 were PT21/28, PT8 and PT32. Similarly, in our study population, PT21/28 and PT8 were commonly reported, in addition to PT1 and PT2. No association was observed between PT and incubation period. With the use of genomics in the characterisation of STEC [21], additional microbiological data are available for future analysis and it may be possible to identify pathogen factors affecting incubation period.

HUS is a known complication of STEC O157 infections. Our study, with 17 of 205 cases reporting HUS with a mean incubation period of 5.1 days, found no significant association with incubation period. A consistent result was reported in a systematic review (Awofisayo-Okuyelu *et al*. Accepted – Epidemiological Reviews) where no significant difference was observed in the incubation period of outbreaks involving HUS cases and those that did not.

Our study has several limitations. The cases we analysed were a small total of all of the cases in the included outbreaks due to missing information. In contrast with cases associated with animal contact, the incubation period could not be calculated for 86% of foodborne-associated outbreaks which had to be excluded. The characteristics of included cases were significantly different from the excluded cases; however, the impact on internal validity is likely to be limited unless those excluded had substantially different incubation periods or different associations between risk factors and incubation period than those included. We had few cases associated with environmental exposure so that we are not able to offer any robust inference on the distribution of incubation period associated with this transmission route.

Additionally, the proportion of cases reporting each symptom was significantly different between included and excluded cases with a higher proportion of bloody diarrhoea being reported amongst the excluded cases. If these excluded cases had systematically different incubation periods, this could result in bias in our estimates of the distribution of incubation periods.

In conclusion, we used IPD collected across outbreaks to estimate the incubation time distribution of STEC. We analysed patient characteristics like age, gender and reported symptoms as well as outbreak characteristics such as mode of transmission and geographical location in order to identify factors associated with the distribution of incubation period. The median incubation period was 4 days and identified associated factors were cases aged 40–59 years and developing bloody diarrhoea. These conclusions are based on a dataset of which 31% of the cases belong to a single outbreak.

Public health professionals should therefore consider the effect of patient and outbreak characteristics on the incubation period, and outbreak investigations should be tailored accordingly to either extend or restrict the investigative period when collecting exposure details. Further research may be required to determine the effect of characteristics that were not examined in this study such as STEC serogroup and genomic data, underlying medical conditions and ongoing medications.

A major benefit to analysing IPD from outbreaks was having patient-level information that might be associated with the distribution of incubation period. However, there were several limitations to the quality of recording of data to support our purposes as might be expected from systems not established for this particular purpose. The detail of information available for analysis depended on how comprehensively the cases answered the questions on the ESQ or how efficiently they were asked by the interviewer. We observed that the question regarding the date of exposure was mostly unanswered, as well as not being explicitly included for every exposure. It was often easier to obtain dates of exposure from cases in outbreaks associated with a location such as farm visits, than it was to obtain dates of exposure from outbreaks associated with purchased food items. For purchased food items, the ESQ records the date the item was purchased and not the date of consumption. Similarly, other patient information which may be useful for our study was not collected at all, such as underlying medical conditions and ongoing medications. Studies such as this one that show the research potential of routine surveillance data may guide future surveillance to ensure that we consider such applied research outcomes prospectively in planning surveillance. Consideration of research applications is likely to inform content and form of data capture as well as consent processes where relevant.

## Supplementary material

For supplementary material accompanying this paper visit https://doi.org/10.1017/S0950268819000451. click here to view supplementary material
