# Supplementary material for: Analysis of individual patient data to describe the incubation period distribution of Shiga-toxin producing Escherichia coli
Source: Epidemiol Infect. 2019 Mar 27;147:e162. doi: 10.1017/S0950268819000451 (PMC6518530; doi:10.1017/S0950268819000451)
Supplement: Supplementary file 1 [file S0950268819000451sup001.docx]

*Epidemiology and Infection*

**Title:** Analysis of individual patient data to describe the incubation period distribution of STEC

**Authors:**

A. Awofisayo-Okuyelu, I. Hall, E. Arnold, L. Byrne, N. McCarthy

**Supplementary Material**

Supplementary Table S1. Characteristics of excluded cases compared with cases included in the study

| **Variable** | **Number of excluded cases**  **(N = 1254)** | **Proportion of cases in excluded group** | **Proportion of cases in included group** | **Chi square for difference in proportion** |
| --- | --- | --- | --- | --- |
| **Age group** |  |  |  | <0.0001 |
| 0 – 4 years | 262 | 20.9 | 33.7 |  |
| 5 – 9 years | 132 | 10.5 | 23.4 |  |
| 10 -19 years | 140 | 11.2 | 11.2 |  |
| 20 - 29 years | 120 | 9.6 | 12.7 |  |
| 30 - 39 years | 219 | 17.5 | 7.8 |  |
| 40 - 59 years | 169 | 13.5 | 4.9 |  |
| 60 - 79 years | 161 | 12.8 | 4.9 |  |
| 80 and above | 51 | 4.1 | 1.4 |  |
| **Gender** |  |  |  | >0.5 |
| Females | 758 | 60.4 | 59 |  |
| Males | 496 | 39.6 | 41 |  |
| **Ethnicity** |  |  |  | <0.0001 |
| White | 1147 | 91.5 | 81 |  |
| Mixed ethnicity | 9 | 0.7 | 1.4 |  |
| Asian/Asian British | 14 | 1.1 | 0 |  |
| Black/Black British | 13 | 1.0 | 0.5 |  |
| Chinese | 2 | 0.2 | 0.5 |  |
| Other | 6 | 0.5 | 0 |  |
| Unknown | 63 | 5.0 | 16.6 |  |
| **Relevant occupation** |  |  |  | >0.5 |
| Food handler | 60 | 4.8 | 3.9 |  |
| Healthcare worker | 75 | 6.0 | 2 |  |
| Childcare workers | 220 | 17.5 | 19.5 |  |
| **Geographical region of patient residence** |  |  |  | <0.0001 |
| East Midlands | 78 | 6.2 | 1.9 |  |
| East of England | 121 | 9.6 | 8.3 |  |
| London | 96 | 7.7 | 15.6 |  |
| North East | 78 | 6.2 | 13.2 |  |
| North West | 217 | 17.3 | 13.2 |  |
| South East | 220 | 17.5 | 21.9 |  |
| South West | 214 | 17.1 | 7.8 |  |
| Wales | 25 | 2.0 | 0.5 |  |
| West Midlands | 95 | 7.6 | 4.4 |  |
| Yorkshire and Humber | 109 | 8.7 | 13.2 |  |
| Unknown | 17 | 1.4 | 0 |  |
| **Reported symptoms** |  |  |  | <0.05 |
| HUS | 61 | 4.9 | 8.3 |  |
| Diarrhoea | 1012 | 80.7 | 93.2 |  |
| Bloody diarrhoea | 968 | 77.2 | 65.9 |  |
| Nausea | 900 | 71.8 | 49.8 |  |
| Vomiting | 904 | 72.1 | 42.4 |  |
| Abdominal pain | 993 | 79.2 | 80 |  |
| Fever | 874 | 69.7 | 27.8 |  |
| **Mode of transmission** |  |  |  | <0.0001 |
| Direct animal contact | 108 | 12.8 | 63.9 |  |
| Environmental exposure | 12 | 1.4 | 4.4 |  |
| Foodborne | 724 | 85.8 | 31.7 |  |
| **Geographical region of outbreak** | Number of outbreaks (N = 95) |  |  | 0.5 |
| East Midlands | 6 | 6.3 | 7.3 |  |
| East of England | 3 | 3.2 | 7.3 |  |
| London | 2 | 2.1 | 0 |  |
| National | 18 | 18.9 | 9.8 |  |
| North East | 7 | 7.4 | 4.9 |  |
| North West | 15 | 15.8 | 17.1 |  |
| South East | 13 | 13.7 | 12.2 |  |
| South West | 13 | 13.7 | 17.1 |  |
| Wales | 1 | 1.1 | 2.4 |  |
| West Midlands | 3 | 3.2 | 2.4 |  |
| Yorkshire and Humber | 11 | 11.6 | 19.5 |  |
| International | 3 | 3.2 | 0 |  |
